# Investigation of facilitative urea transporters in the human gastrointestinal tract

**DOI:** 10.14814/phy2.13826

**Published:** 2018-08-12

**Authors:** Caragh Walpole, Alison McGrane, Hashemeya Al‐mousawi, Desmond Winter, Alan Baird, Gavin Stewart

**Affiliations:** ^1^ School of Biology & Environmental Science University College Dublin Dublin Ireland; ^2^ Institute for Clinical Outcomes Research and Education St. Vincent's University Hospital Dublin Ireland; ^3^ College of Life Sciences Conway Institute of Biomedical and Biomolecular Science Dublin Ireland

**Keywords:** Gastrointestinal tract, human, UT‐A, UT‐B

## Abstract

The symbiotic relationship between humans and their intestinal microbiome is supported by urea nitrogen salvaging. Previous studies have shown that colonic UT‐B urea transporters play a significant role in this important physiological process. This current study investigated UT‐A and UT‐B urea transporter expression along the human gastrointestinal tract. Initial end‐point PCR experiments determined that UT‐A RNA was predominantly expressed in the small intestine, while UT‐B RNA was expressed in stomach, small intestine, and colon. Using western blotting experiments, a strong 40–60 kDa UT‐B signal was found to be abundant in both ileum and colon. Importantly, this signal was deglycosylated by PNGaseF enzyme treatment to a core protein of 30 kDa in both tissues. Further immunolocalization studies revealed UT‐B transporter proteins were present at the apical membrane of the villi in the ileum, but predominantly at the basolateral membrane of the colonic surface epithelial cells. Finally, a blind scoring immunolocalization study suggested that there was no significant difference in UT‐B abundance throughout the colon (NS, ANOVA,* N* = 5–21). In conclusion, this current study suggested UT‐B to be the main human intestinal urea transporter. Intriguingly, these data suggested that the same UT‐B isoform was present in all intestinal epithelial cells, but that the precise cellular location varied.

## Introduction

Mammalian facilitative urea transporters (UTs) enable rapid movement of urea across plasma cell membranes and are encoded by either the Slc14a1 (UT‐B) or Slc14a2 (UT‐A) genes (Stewart 2011). These UTs play crucial roles in both the urinary concentrating mechanism in the kidney (Fenton et al. 2004) and the urea nitrogen salvaging (UNS) process in the gastrointestinal tract (Stewart 2011). Crucially, UNS supports the symbiotic relationship between mammals and their intestinal microbiome by providing a valuable source of nitrogen that enables continual microbial growth (Stewart and Smith 2005). Over the last 15 years, there has been an exponential increase in our appreciation of the role the human colonic microbiome plays in health and disease – for example in inflammatory bowel diseases, such as ulcerative colitis (Shen et al. 2018), and in colon cancer (Zou et al. 2018). As a result, a better understanding of the UNS process that helps support the colonic microbiome is now urgently required.

Previous studies have shown facilitative UT‐B urea transporters to be present within gastrointestinal tissues of many mammalian species – including humans (Inoue et al. 2004), mice (Lucien et al. 2005), rats (Inoue et al. 2005), cows (Coyle et al. 2016), sheep (Ludden et al. 2009), and goats (Lu et al. 2015). Importantly, functional UT‐mediated trans‐epithelial urea transport has been reported in many of these tissues, such as the bovine rumen (Stewart et al. 2005), and the rat colon (Collins et al. 2011). In non‐human species, it is also now known that the role of these UT‐B transporters in UNS can be regulated by dietary intake, pH or urea levels (Inoue et al. 2005; Simmons et al. 2009; Lu et al. 2015). However, little is currently known regarding the precise nature of UTs along the full length of the human intestinal tract.

After the initial report of UT‐B expression in the human colon twenty years ago (Ritzhaupt et al. 1998), relatively little progress has been made. Our previous work has shown that glycosylated UT‐B transporters, situated in the upper epithelial layers lining the colon, appear to be responsible for the increased trans‐epithelial urea transport detected in the right (ascending) human colon (Collins et al. 2010). In addition, we have recently shown that the UT‐A6 transporter initially identified in human colon (Smith et al. 2004) is differentially expressed along the human gastrointestinal tract (right colon > left colon > small intestine ≫ stomach) (McGrane and Stewart 2016). Our studies also revealed that UT‐A6 expression in the caco‐2 intestinal cell line was highly regulated by changes in external osmolality (McGrane and Stewart 2016). However, little else has been documented regarding other human intestinal urea transporters, either UT‐A or UT‐B, and their potentially vital role in supporting our microbiome. The aim of this current study was therefore to better characterize the expression, abundance, and cellular localization of facilitative urea transporters along the human gastrointestinal tract.

## Methods

### End‐point RT‐PCR

Using purchased human tissue RNA samples (AMS Biotechnology, UK), cDNA preparation was performed using a SensiFast cDNA synthesis kit (Bioline, UK). Resulting cDNA samples underwent PCR amplification with a Go‐*Taq* polymerase enzyme (Promega, Ireland), using UT‐A, UT‐B or actin primers (see Table 1).

**Table 1:** Sequences of all end‐point PCR primers sets, plus number of cycles, denaturing temperature, and the expected product sizes

Primer	Sequence	Number of cycles	Denaturing temperature (°C)	Product size (bp)
UT‐A F5	5′ – GTGGCTTCTGTTTCCTGTGACCTT – 3′	35	60	**UT‐A1/3**
UT‐A R10	5′ – CCTCGGGGTAGGTGACTTTGCTGAGT – 3′	35	60	F5/R10 = 682
UT‐A F23	5′ – CTGTTCATATCCTCACCTCTCATTTG – 3′	35	60	**UT‐A1/2**
UT‐A R25	5′ – CACAGATAACATGTTAGCCAGGGC – 3′	35	60	F23/R25 = 252
UT‐B F1	5′ – GCCAGGAAGCCAGCTAGAGTGGTC – 3′	35	60	**UT‐B1 or UT‐B2**
UT‐B R5	5′– CTGTCCTGGCTGAGCAAGAGG – 3′	35	60	F1/R5 = 450 or 600
UT‐B F4	5′ – CATCTTGCCAACCAGCTTAAAG – 3′	35	60	**UT‐B**
UT‐B F6	5′ – GGTGGGAGTACTCATGGCTGTCTTTTC – 3′	35	60	F4/R5 = 219
UT‐B R6	5′ – GTAACAGCCACCAGAAATAGTC – 3′	35	60	F4/R6 = 320
UT‐B R10	5′ ‐ GCCATAAAGTTTGCCATGCCG ‐ 3′	35	60	F4/R10 = 870 F6/R10 = 609
Actin_F	5′ – GTGCTGTCTGGCACCACCAT – 3′	30	55	**Actin**
Actin_R	5′ – CCTGTAACAACGCATCTCATAT – 3′	30	55	514

KEY: F = forward primer; R = reverse primer; “F5” = forward primer targeted to exon 5 etc.

Cycling parameters were initial denaturation at 94°C for 2 min, followed by 30 or 35 cycles at 94°C for 30 sec, 55 or 60°C for 30 sec, and 72°C for 30 sec (see Table 1). The final extension was at 72°C for 5 min. Identity of PCR products were confirmed through direct sequencing (Eurofins MWG, Operon, Germany).

### Antibodies

To study UT‐B transporter protein, the previously characterized C‐terminal hUT‐Bc19 polyclonal antibodies were utilized (Walpole et al. 2014). Commercially available antibodies were used in an attempt to detect UT‐A transporters (NBP1‐44375, Novus Biologicals). Both of these urea transporter antibodies were used in connection with horseradish peroxidase‐conjugated secondary anti‐rabbit IgG antibody (65‐6120, Invitrogen).

### Tissue source and ethical approval

Human ileum and colon samples were obtained at surgical resection for carcinoma. The normal histological appearance of tissues was confirmed by routine pathological examination of samples during resection. Tissues from the resection margins were immediately transferred to the laboratory in preoxygenated Krebs‐Henseleit solution (composition in mmol/L: 118 NaCl, 4.7 KCl, 2.5 CaCl_2_, 1.2 MgSO_4_, 1.2 KH_2_PO_4_, 25 NaHCO_3_ and 11.1 d‐glucose, pH 7.4). St. Vincent's University Hospital Institutional Review Board approval, including informed patient consent, was granted for this study. [Note – Human bladder, liver, and red blood cells protein samples were commercially purchased from AMS Biotechnology, UK.]

### Western blotting

Tissue samples were initially homogenized with an automated homogenizer and a specifically prepared ice‐cold buffer (300 mmol/L mannitol, 12 mmol/L HEPES, pH 7.6). Centrifugation of these whole cell homogenates was first performed at 2500*g* for 5 min, then the resulting supernatant was spun at 16,900*g* for 30 min at 4°C. The pellet formed was then re‐suspended in fresh homogenization buffer, resulting in a membrane‐enriched protein sample. For deglycosylation experiments, protein samples were incubated with and without peptide‐N‐glycosidase F enzyme for 1 h at 37°C. For loading samples onto gels, 2X reducing Laemmli sample buffer [5% SDS, 25% glycerol, 0.32 mol/L Tris (pH 6.8), bromophenol blue, and 5% beta‐mercaptoethanol] was added to all protein samples at a ratio of 1:1 and heated at 70°C for 15 min. SDS‐PAGE was performed on 4–16% gradient polyacrylamide by loading approximately 20 *μ*g protein/lane. After transfer to nitrocellulose membranes, immunoblots were probed for 16 h at room temperature in 1:1000 hUT‐Bc19 or UT‐A antibodies. After incubation in primary antibody, immunoblots were washed and then probed with 1:5000 horseradish peroxidase‐conjugated anti‐rabbit antibody for 1 h at room temperature. After further washing, detection of protein was performed using Western Lightning Plus ECL reagents (Perkin‐Elmer) and a LAS‐4000 Image Reader (Fujifilm).

### Immunolocalization

Human ileum tissue sections (10 *μ*m in thickness) were purchased from AMS Biotechnology (UK), while colonic microarray slides were obtained from Novus Biologicals (UK). After Neo‐clear^®^ TM treatment and rehydration of these sections in a descending series of ethanol concentrations (100–70%), endogenous peroxidase was blocked by incubating sections for 30 min in 3% hydrogen peroxide in methanol. Antigen retrieval was performed by boiling sections for 1 min in a solution containing 25 mmol/L TRIS‐HCl (pH 8.0) and 10 mmol/L EDTA before an overnight incubation at 4°C with 1:500 dilution of hUT‐Bc19 in 0.1% BSA and 0.3% Triton X‐100 in PBS. Immunolabeling was visualized with a 1 h incubation in 1:500 horseradish peroxidase‐conjugated anti‐rabbit secondary antibody, followed by an incubation with diaminobenzidine and counterstaining with hematoxylin. An ascending series of ethanol concentrations (70–100%) was then used to dehydrate the stained sections and subsequently treated with Neo‐clear^®^. Finally, coverslips were mounted using Eukitt mounting medium, and slides were stored at room temperature. Detailed images of sections were obtained using a Labophot 2 microscope (Nikon), a Micropublisher‐RTV‐3.3 Digital camera (Q Imaging), and Image‐Pro Plus image‐analysis software (MediaCybernetics).

## Results

**Figure 1:**
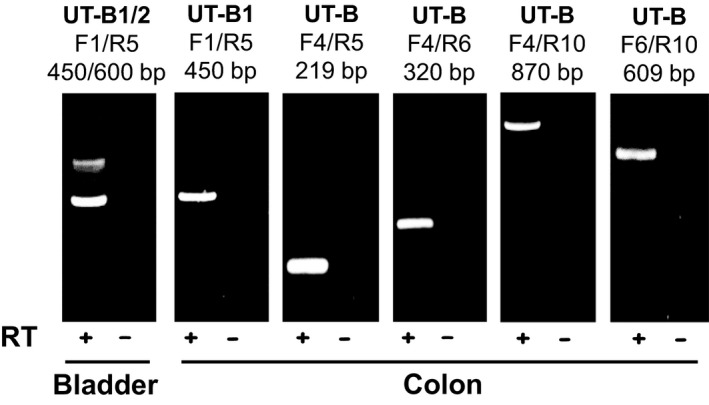
End‐point PCR experiments investigating UT‐B expression in human colon. Using cDNA derived from pooled total RNA adult colon samples (*N* = 5), strong signals were detected with all the UT‐B primer sets tested. In each case, the predicted size product for UT‐B1 transcripts was produced, with no other strong signals evident. For example, the F1/R5 primers detected the 450 bp product expected with UT‐B1 in colon but not the 600 bp UT‐B2 signal, which was detected in human adult bladder. Key: RT = reverse transcriptase; + = RT present; − = RT absent.

Initial RT‐PCR experiments were performed to investigate UT‐B expression in the human colon using primers designed against different exons in the UT‐B gene (see Table 1). The F1/R5 primer set, targeted to exons 1 and 5 in the N‐terminal region of UT‐B, only detected a strong signal at the 450 bp expected size for UT‐B1 in the colon and not at the 600 bp expected size for UT‐B2 (see Fig. 1). In contrast, both 450 and 600 bp signals were detected in the human bladder (see Fig. 1). All other UT‐B primer sets, designed to exons in other regions of the UT‐B gene, also detected a strong signal at the expected size for UT‐B1 – namely 219 bp for F4/R5, 320 bp for F4/R6, 870 bp for F4/R10 and 609 bp for F6/R10 (see Fig. 1). The identity of all five PCR products was confirmed to be human UT‐B by direct sequencing, with no deletions or other mutations being detected (data not shown).

**Figure 2:**
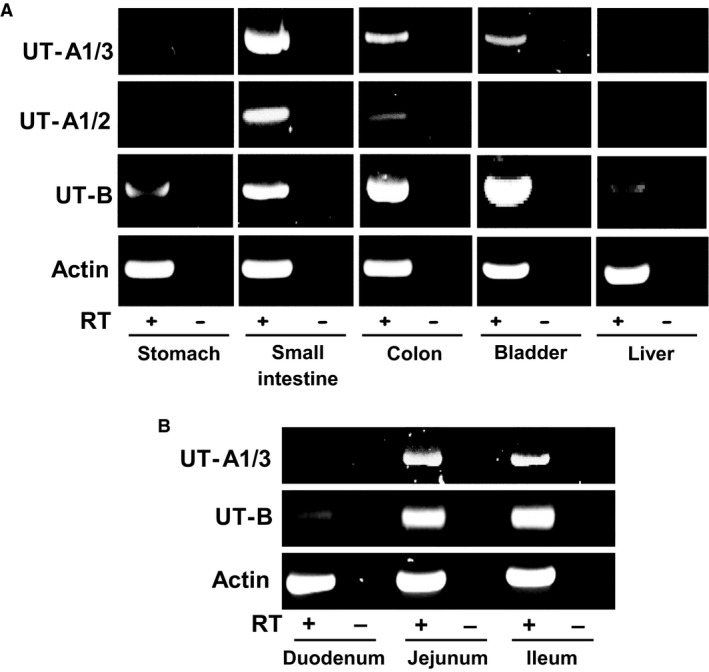
End‐point PCR experiments investigating UT‐A and UT‐B expression in various human tissues. (A) Using cDNA derived from pooled total RNA adult samples (*N* = 5), strong signals were detected using UT‐A1/3, UT‐A1/2 and UT‐B (F6/R10) primer sets. Both UT‐A primer sets detected strong signals in small intestine and weaker signals in colon and bladder. UT‐B was strongly detected in small intestine, colon, and bladder, with only weak signals in stomach and liver. In contrast, control experiments using actin primers gave strong signals with +RT reactions for all five tissues. (B) Using cDNA derived total RNA adult samples, urea transporter expression in distinct regions of the small intestine was also investigated. UT‐A expression was detected in both jejunum and ileum, but not duodenum. Similarly, strong UT‐B signals were detected in jejunum and ileum, with only weak expression in duodenum. Control experiments with actin showed strong signals in +RT samples of all three tissues. Key: RT = reverse transcriptase; + = RT present; − = RT absent.

Next, additional primer sets (see Table 1) were utilized to investigate the expression of UT‐A, UT‐B, and actin in various human tissues, including the gastrointestinal tract (see Fig. 2A). The UT‐A1/3 primer set (UT‐A F5/R10) was designed to detect UT‐A1 and UT‐A3 transcripts. These primers produced a strong signal in small intestine, weak signals in colon and bladder, and no signals in stomach and liver. Sequencing of the small intestine product confirmed this is to be a 100% match for UT‐A1, and hence UT‐A3 (data not shown). The UT‐A1/2 primer set (UT‐A F23/R25) was designed to detect UT‐A1 and UT‐A2 transcripts. These primers again detected a strong signal in small intestine, a weak signal in colon, and no signals in stomach, bladder, and liver (see Fig. 2A). Once more, sequencing of the small intestine product confirmed this is to be a 100% match for UT‐A1, and hence UT‐A2 (data not shown). Alternatively, UT‐B primers (F6/R10) detected strong signals in small intestine, colon, and bladder, with weak signals in stomach and liver. Importantly, the positive control actin primers detected strong signals for all five tissues (see Fig. 2A). Further analysis of the different subsections of the small intestine showed there was no UT‐A signal and only a very weak UT‐B signal in duodenum (see Fig. 2B). In contrast, strong signals for UT‐A and UT‐B were detected in both jejunum and ileum (see Fig. 2B).

**Figure 3:**
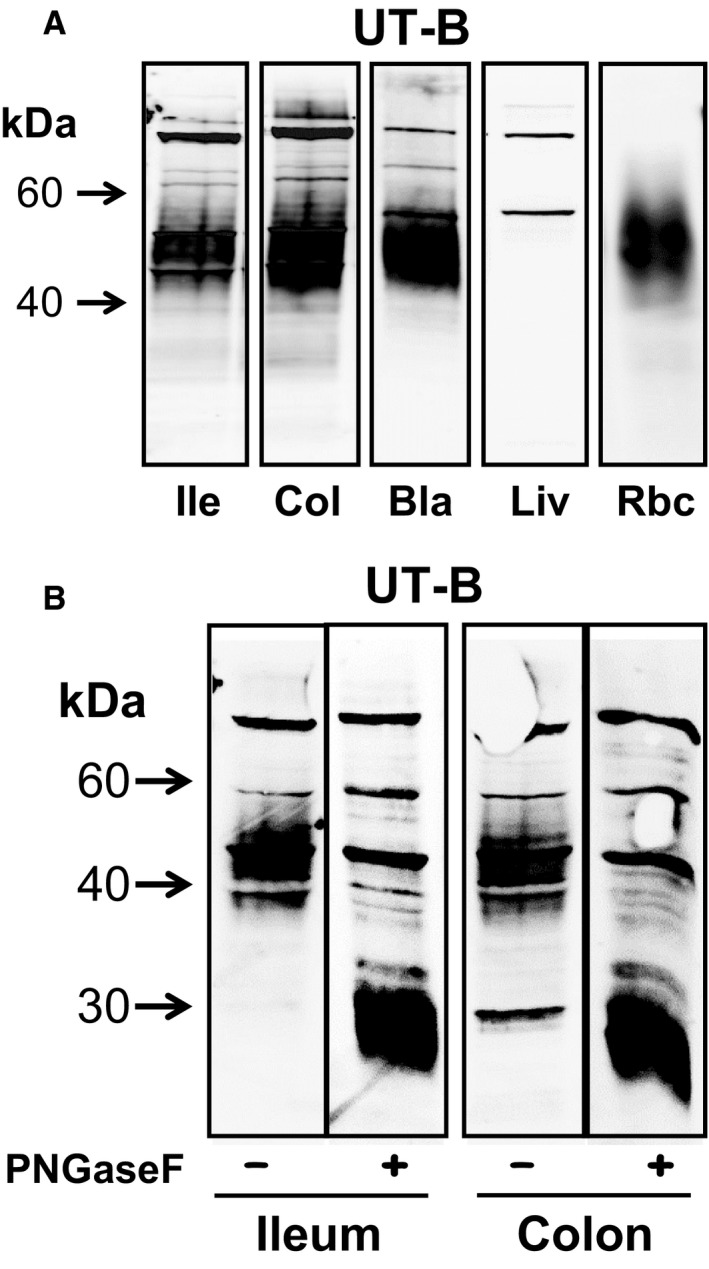
Western blotting experiments investigating UT‐B protein abundance in various tissues. (A) Using membrane‐enriched protein samples, hUTBc19 antibodies detected strong, smeared 40–60 kDa UT‐B signals in ileum, colon, bladder, and red blood cells. In contrast, no such signal was detected in liver, where only weak, tight bands at ~50 and ~100 kDa were detected. (B) The effect of PNGaseF treatment was investigated on the UT‐B signals in ileum and colon. In both tissues, PNGaseF deglycosylated the 40–60 kDa signal to an unglycosylated core protein at ~30 kDa. Key: Ile = ileum; Col = colon; Bla = bladder, Liv = liver; Rbc = red blood cells; PNGaseF = PNGaseF enzyme; + = PNGaseF treated; − = untreated.

In an attempt to determine UT‐A protein abundance in human ileum and colon, western blotting experiments were performed with commercially available polyclonal UT‐A antibodies. Unfortunately, our experiments failed to provide any evidence that these antibodies were viable (data not shown). Further western blotting experiments were therefore performed to investigate UT‐B protein abundance. Using the previously characterized hUTBc19 polyclonal antibodies (Walpole et al. 2014) and membrane‐enriched protein samples, we detected strong, smeared 40–60 kDa UT‐B signals in ileum, colon, bladder, and red blood cells (see Fig. 3A). In contrast, no such signal was detected in liver, where only weak, tight bands at 50 and 100 kDa were detected. The effect of pre‐incubation with PNGaseF deglycosylating enzyme was then investigated on the UT‐B signals in ileum and colon. In both tissues, treatment with PNGaseF deglycosylated the original 40–60 kDa smeared signal to an unglycosylated core protein at 30 kDa (see Fig. 3B).

**Figure 4:**
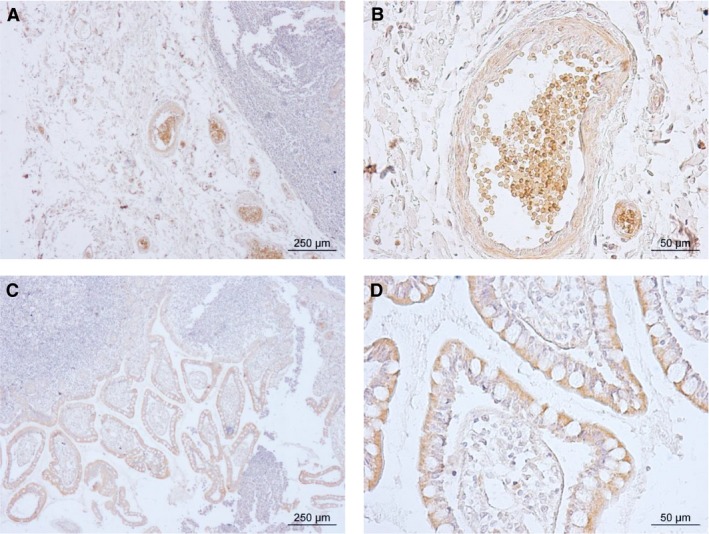
Immunolocalization showing UT‐B staining of a 10 *μ*m transverse section of human ileum. Strong staining of UT‐B was detected in red blood cells situated within blood vessels – (A) ×4 magnification, and (B) ×20 magnification. Strong UT‐B staining was also observed in the apical region of epithelial cells of the intestinal villi – (C) ×4 magnification, and (D) ×20 magnification.

**Figure 5:**
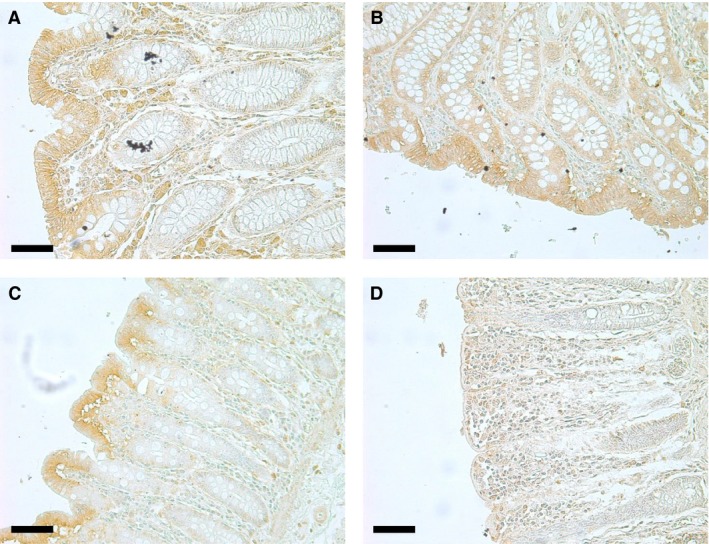
Immunolocalization showing UT‐B staining of 10 *μ*m transverse sections of different regions of the human colon. (A) Ascending colon (×20 magnification), (B) Tranverse colon (×20 magnification, (C) Descending colon (×20 magnification), and (D) Sigmoid colon (×20 magnification). All scale bars represent 50*μ*m.

In order to investigate the cellular localization of the UT‐B protein detected in the human gastrointestinal tract, an immunolocalization study using the hUTBc19 antibodies was performed. Using purchased 10 *μ*m thick transverse sections, strong UT‐B signals were detected in the ileum in two distinct locations (see Fig. 4). First, as was predicted, a strong UT‐B signal was observed in the blood vessels (see Fig. 4A) and higher magnification images confirmed this to be staining of the red blood cell plasma membranes (see Fig. 4B). Second, strong UT‐B staining was also observed in the intestinal villi (see Fig. 4C) and higher magnification revealed that this staining was situated in the apical regions of the epithelial cells lining the outside of the villi (see Fig. 4D). Next, using multiple sections, UT‐B localization was determined in all the regions of the human colon (see Fig. 5). In ascending colon sections, strong staining was often observed in the upper regions of the epithelial layer (see Fig. 5A). This staining was present in both surface epithelial cells and also in the interstitial tissue. In transverse colon sections, strong UT‐B staining was mainly present in the surface epithelial cells (see Fig. 5B). Similarly, when strong UT‐B signals were detected in descending colon sections, they were restricted to basolateral regions of the surface epithelial cells (see Fig. 5C). In comparison, generally less staining was observed in sigmoid colon sections, with no clear UT‐B signals (see Fig. 5D). Finally, scoring of epithelial UT‐B immunostaining in multiple sections of the different colonic regions was performed in a blind study (see Table 2). However, although a trend was observed, these data showed that there was statistically no significant differences in UT‐B staining across different sections of the colon (NS, ANOVA).

**Table 2:** Summary of the UT‐B immunostaining scores from different human colonic regions

	UT‐B immunolocalization score
Ascending colon (*N* = 16)	1.38 ± 0.20
Transverse colon (*N* = 7)	1.57 ± 0.20
Descending colon (*N* = 5)	1.20 ± 0.58
Sigmoid colon (*N* = 21)	0.95 ± 0.15
	*P* = 0.2813

“Blind” scoring of the numerous UT‐B stained colonic sections was performed and the mean scores were then calculated for each colonic region (mean ± SE). Scoring key: 0 = no staining; 1 = weak UT‐B staining; 2 = moderate UT‐B staining; 3 = strong UT‐B staining.

## Discussion

From our initial RT‐PCR experiments, we were able to determine that a number of urea transporter transcripts were present in different regions of the human gastrointestinal tract. First, it was confirmed that UT‐B1, and not UT‐B2, was the main transcript present in human colon (see Fig. 1). This finding appears to rule out the previous suggestion that both may play a role in the human colon (Collins et al. 2010). Next, variable levels of expression for UT‐A and UT‐B were detected in the gastrointestinal tissues investigated (see Fig. 2A and B). In both stomach and duodenum, only weak signals for UT‐B were present. Surprisingly, this was not the case in jejunum and ileum, which had high RNA expression levels for both UT‐A and UT‐B. By comparison, the colon expressed high levels of UT‐B but appeared to display only very limited UT‐A expression (see Fig. 2A). Interestingly, the weak expression of UT‐B and absence of recognized UT‐A transcripts in the human liver are similar to previous findings for the mouse liver, in which both UT‐A and AQP‐9 transporters were shown to be functionally significant (Jelen et al. 2012). The complete absence of UT‐B protein in human liver (see Fig. 3A) seems to rule out these transporters contributing to functional urea transport pathway in this major urea‐producing organ, the precise details of which remain strangely elusive to researchers.

Using western blotting, protein abundance was investigated but unfortunately the UT‐A antibodies utilized in this study were found to be non‐viable (data not shown). As a result, no clear conclusions can be made regarding the presence or otherwise of UT‐A protein in the human gastrointestinal tract. In contrast, the well‐characterized hUTBc19 antibodies detected strong 40–60 kDa smeared UT‐B signals in both ileum and colon – as well as in the positive controls of bladder (Walpole et al. 2014) and red blood cells (Timmer et al. 2001). The ileum and colon signals were completely deglycosylated to a 30 kDa protein (see Fig. 3B). Intriguingly, this is exactly the same size as we reported for UT‐B1 protein in human bladder and rat kidney using the same hUTBc19 antibodies (Walpole et al. 2014). It is also the exact same size we previously reported for unglycosylated human colon UT‐B protein, using different UT‐B antibodies (Collins et al. 2010), and that other researchers reported for deglycosylated UT‐B1 in human red blood cells (Timmer et al. 2001). This 30 kDa size is shorter than the predicted size for a 389 amino acid long protein like UT‐B1 (~40 kDa), and this fact implies that an N‐terminal truncation event may well be involved in the standard cellular processing of functional UT‐B1. It has long been known that two cysteine residues in the N‐terminal of UT‐B are essential for plasma membrane addressing (Lucien et al. 2002), but perhaps the N‐terminal is cleaved after this event and is not required for urea transport function. Further studies using N‐terminal targeted UT‐B antibodies are required to elucidate whether this hypothesis is indeed correct.

**Figure 6:**
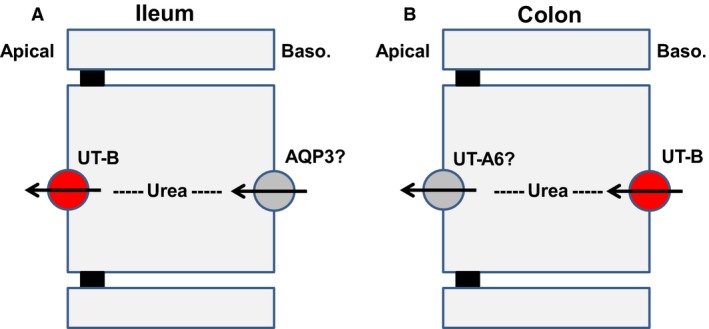
Schematic presentation of potential trans‐epithelial urea transport pathways in human gastrointestinal tissue – (A) Ileum, and (B) Colon. Confirmed UT‐B transporter location shown in red, while suggested potential other transporters that could be involved are shown in gray.

With no viable UT‐A antibodies available, immunolocalization studies investigated the cellular location of UT‐B protein in the gastrointestinal tract. Human ileum contained UT‐B staining in the intestinal epithelial cells lining the villi and within the red blood cells in the blood vessels (see Fig. 4A–D). Within the villi epithelial cells, UT‐B staining appeared to be particularly prevalent in apical regions (see Fig. 4D). This apical membrane location suggest UT‐B in the ileum is functionally involved in trans‐epithelial urea transport, presumably the secretion of urea into the gastrointestinal tract. The fact that UT‐B protein is fully glycosylated (see Fig. 3B) is another indication that it is functional in the human ileum (Collins et al. 2010). No basolateral UT‐B staining was detected in the ileum, so what could the transport mechanism be across this membrane? Interestingly, functional studies in the bovine rumen – a tissue that displays very high levels of UT‐B (Stewart et al. 2005) – have shown that trans‐epithelial urea transport also involves aquaglyceroporins, such as AQP3 (Walpole et al. 2015). Since it has also been reported that AQP3 is strongly basolateral in the human ileum (Mobasheri et al. 2004), we suggest that AQP3 is a strong candidate for the mechanism by which urea could cross the ileal basolateral membrane (see Fig. 6A). Overall, it is tempting to speculate that trans‐epithelial secretion of urea into the human ileum could be very useful to the UNS process – as it would ensure the large bacterial populations present in the right/ascending colon were well supplied with the urea to use as a nitrogen source for growth. Interestingly, a previous study reported that UT‐B in the rat small intestine was also located in the epithelium of ileal mucosa (Inoue et al. 2005).

Our results for UT‐B staining across numerous colonic sections suggested that UT‐B is found throughout the human colon (Fig. 5). The UT‐B staining was predominantly in the colonic surface epithelial cells at or near the basolateral membrane (Fig. 55A‐C). In contrast to the UT‐B staining in the ileum (Fig. 4D), there appeared minimal apical UT‐B in colonic epithelial cells. Further studies are therefore still required to determine if another transporter facilitates urea movement across this membrane – with UT‐A6 (McGrane and Stewart 2016) or an apical aquaglyceroporin (Walpole et al. 2014) being among the potential candidates. Currently, we propose that the most likely candidate is UT‐A6 (Fig. 6B), due to the fact that it's expression level varies across in different colonic regions (McGrane and Stewart 2016) in a manner that matches functional trans‐epithelial urea transport (Collins et al. 2010) that is, UT‐A6 expression and urea transport are both higher in the right colon than the left colon. However, until UT‐A6 protein detection can be successfully performed, this hypothesis will remain untested.

Although there was a trend for stronger staining in the right colon, for example in the ascending colon, there was no significant difference in the blind scoring study performed (Table 2). This is similar to our previous study that suggested the total amount of UT‐B protein does not vary much between the right and left colon (Collins et al. 2010). In this earlier study, we reported that it was differences in the level of UT‐B glycosylation, and hence function, that appeared responsible for the differences we observed in the actual colonic trans‐epithelial transport (Collins et al. 2010). These findings contrast with our recent finding for another colonic transporter involved in the symbiotic relationship between human colonic cells and the colonic microbiome – namely the short chain fatty acid transporter MCT1 – in which transporter abundance was significantly reduced in the sigmoid colon (Al‐mousawi et al. 2016). Overall, these studies highlight the importance of investigating (i) all regions of the human colon and (ii) using multiple research techniques (i.e., PCR, western blotting, immunolocalization, and, if possible, functional analysis) if you are attempting to understand the physiological role of any colonic transporter.

So, what is the significance of these current findings regarding urea transporters in the human gastrointestinal tract? A number of studies have suggested potential links between UT‐B transporter function and human disease. UT‐B is highly expressed in human urothelial cells (Walpole et al. 2014) and UT‐B allelic variation has been shown to potentially affect risk of bladder cancer (Garcia‐Closas et al. 2011; Rafnar et al. 2011). Indeed, UT‐B expression is known to be downregulated in bladder urothelial cancer (Li et al. 2014; Hou et al. 2017) and a mutated UT‐B transporter has now also been identified in this disease (Hou et al. 2017). Interestingly, one study investigating prostate cancer suggests UT‐B may play a role in this disease too (Vaarala et al. 2012). In contrast, a recent study found no link between UT‐B and human kidney disease (Capriolli et al. 2017). The potential influence of UT‐B variation on gastrointestinal diseases has yet to be investigated, but should now be as a matter of some urgency.

In conclusion, this study has determined that UT‐B urea transporter protein is highly abundant in various regions of the human gastrointestinal tract. In contrast, UT‐A transporters expressed at the RNA level are predominantly in the small intestine. The core UT‐B protein of 30 kDa is glycosylated to 40–60 kDa and its’ cellular location appears to vary between the ileum and colon. Further investigations are now required to determine exactly how these intestinal UT‐B transporters are regulated, as well as their potential role in inflammatory bowel diseases.

## Conflict of Interest

None declared.
